# An Update on Advances in Hypopituitarism: Etiology, Diagnosis, and Current Management

**DOI:** 10.3390/jcm13206161

**Published:** 2024-10-16

**Authors:** Pedro Iglesias

**Affiliations:** 1 Department of Endocrinology and Nutrition, Hospital Universitario Puerta de Hierro Majadahonda, Calle Joaquín Rodrigo, 1, 28222 Majadahonda, Madrid, Spain; piglo65@gmail.com; 2 Instituto de Investigación Sanitaria Puerta de Hierro Segovia de Arana, 28222 Majadahonda, Madrid, Spain

**Keywords:** hypopituitarism, hormone replacement therapy, corticotropin, thyrotropin, gonadotropins, growth hormone, prolactin, diagnosis and treatment

## Abstract

This article provides an updated review of hypopituitarism (HP), an endocrine disorder characterized by a deficiency of one or more pituitary hormones. The various etiologies are reviewed, including pituitary neuroendocrine tumors (PitNETs), hypothalamic lesions, genetic mutations, and acquired factors such as head trauma, medications, neoplasms, and infiltrative diseases. It is noted that PitNETs are responsible for approximately half of the cases in adults, whereas in children the causes are predominantly congenital. Diagnosis is based on clinical evaluation and hormonal testing, with identification of the specific hormonal deficiencies essential for effective treatment. Laboratory tests present challenges and limitations that must be understood and addressed. Hormone replacement therapy is the mainstay of treatment, significantly improving patients’ quality of life. It is important to know the possible interactions between hormone replacement therapies in HP. Recent advances in understanding the pathophysiology of HP and the importance of a multidisciplinary approach to the management of associated complications are discussed. This article emphasizes the need for comprehensive evaluation and continuous follow-up to optimize outcomes in patients with HP and highlights the importance of ongoing research to improve diagnostic and treatment strategies.

## 1. Introduction

The anterior pituitary produces six hormones (adrenocorticotropin, ACTH; thyrotropin, TSH; the gonadotropins (Gn) follicle-stimulating hormone, FSH, and luteinizing hormone, LH; growth hormone (somatotropin), GH; and prolactin, PRL) that regulate five hormonal axes (corticotropic, thyrotropic, gonadotropic, somatotropic, and lactotropic axes), while the posterior pituitary releases two hypothalamic hormones (antidiuretic hormone, ADH or arginine-vasopressin, AVP, and oxytocin).

Hypopituitarism (HP), or pituitary insufficiency, is a clinical syndrome that develops as a result of hyposecretion of one or more hormones from the anterior pituitary, with or without involvement of the posterior pituitary. Hormone deficiency can be isolated, affecting a single axis, or combined, affecting several axes. The term “panhypopituitarism” (panHP) is used when there is a global deficiency affecting all hormonal axes.

HP can originate from an intrinsic inability of the anterior pituitary to produce hormones or from an insufficient effect of hypothalamic trophic hormones (ACTH-releasing hormone, CRH; TSH-releasing hormone, TRH; Gn-releasing hormone, GnRH; and GH-releasing hormone, GHRH) on adenohypophyseal hormones.

HP is a rare disorder, with an estimated incidence in the adult population of 2.1–4.2 cases/100,000 inhabitants/year and an estimated prevalence of 37.5–45.5 cases/100,000 inhabitants [1,2]. In children, HP has an estimated prevalence of between 1/16,000 and 1/26,000 individuals [3]. Although it is not a common disease, its prevalence is increasing, mainly due to brain damage after traumatic brain injury (TBI) and the increase in diagnostic tests (hormonal evaluation and imaging tests). Analysis of more than 1300 patients with HP shows that the proportion of men is slightly higher than that of women (52.7% vs. 47.3%), and the mean age at diagnosis is approximately 47 years (Table 1).

The clinical importance of HP lies in its potential to negatively influence quality of life and to cause a wide range of symptoms and complications as well as its association with a high impact on morbidity and mortality, not only in the long term, but also in acute clinical situations requiring hospitalization [2,6,7]. The complexity of diagnosing HP, especially in cases with atypical presentations or underlying conditions, highlights the importance of comprehensive evaluation and individualized hormone replacement therapy to improve patient outcomes and quality of life [8,9].

In recent years, there have been significant advances in both the understanding and management of HP. This review provides an update on recent advances in the etiology, diagnosis, and treatment of this complex endocrine disorder. Newly discovered underlying causes contributing to pituitary insufficiency, newer and more accurate diagnostic procedures, and current therapeutic approaches used in the management of HP are reviewed.

## 2. Update and Advances in the Etiology of Hypopituitarism

**Figure 1:**
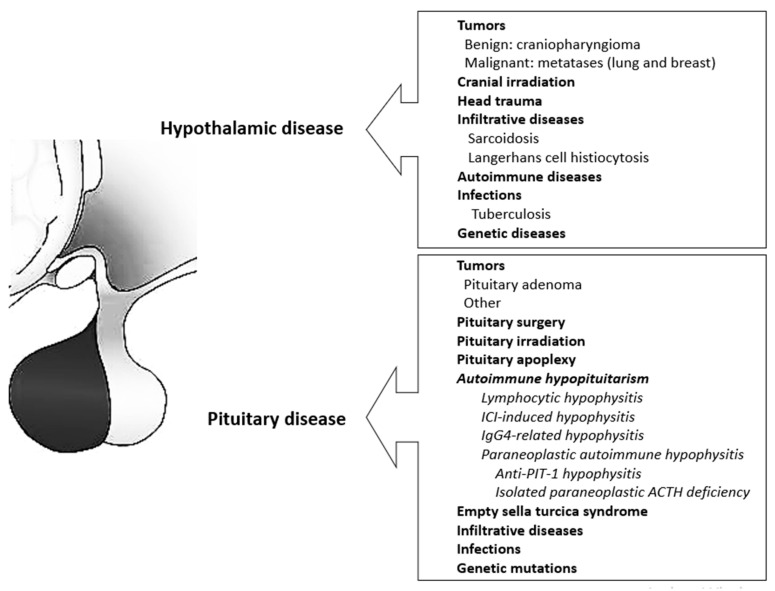
Diagram showing the main etiologies of hypopituitarism.

HP may develop as a consequence of diseases affecting the pituitary gland, the hypothalamus, or both (Figure 1).

In adults, HP due to pituitary neuroendocrine tumors (PitNETs) or their treatment accounts for approximately half of the cases (49.9%), while non-tumor etiologies and non-PitNET tumors are less common, at 42.3% and 7.7%, respectively (Table 1).

In children, PitNETs are rare, and the main causes of HP are congenital diseases due to abnormal pituitary development, genetic mutations (such as PROP1 and PIT1), chromosomal abnormalities such as deletions in chromosome 18, and acquired factors such as tumors (mainly tumors related to alterations in embryogenesis such as craniopharyngiomas and dysgerminomas) or cranial irradiation [3,10,11,12,13]. In addition, TBI, autoimmune and inflammatory diseases, and the use of immune checkpoint inhibitors (ICI) for cancer treatment can also cause HP in children [14].

A variety of hypothalamic lesions may affect the secretion of one or more of the hypothalamic hormones that regulate the pituitary hormones. In contrast to exclusively pituitary lesions, hypothalamic lesions may be associated with impaired AVP secretion and hyperprolactinemia due to a lack of PRL inhibitory control. Several hypothalamic lesions associated with different diseases and/or their treatments can lead to hypothalamic syndrome. It is characterized by different degrees of HP, severe morbid obesity, memory impairment, reduced impulse control, alterations in thermoregulation and thirst control, and increased risk of cardiovascular and metabolic disease [15].

### 2.1. Tumors

The mechanisms by which a pituitary tumor can cause HP are as follows: (1) direct compression of normal pituitary tissue, damaging pituitary cells and reducing their ability to produce hormones; (2) interference with blood supply by tumor compression of blood vessels supplying the pituitary; (3) local inflammation and mass effect, causing edema and changes in the pituitary microenvironment, disrupting normal pituitary function; and (4) damage to hypothalamic–pituitary pathways, disrupting hormonal signaling from the hypothalamus to the pituitary. This may result in decreased release of regulatory hormones from the hypothalamus and, consequently, decreased secretion of pituitary hormones. In adults, the most common sellar masses are PitNETs, meningiomas, craniopharyngiomas, aneurysms, and gliomas.

**Figure 2:**
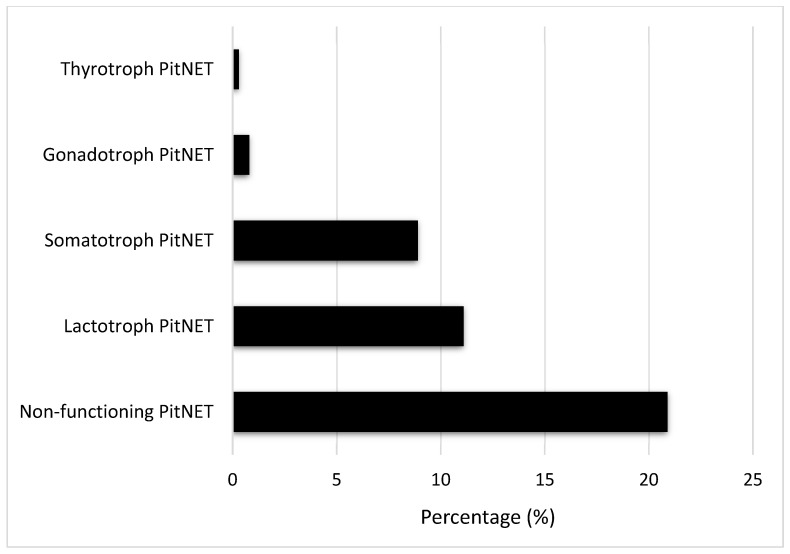
Percentage distribution of the different histologic types of pituitary neuroendocrine tumors (PitNETs) associated with hypopituitarism (adapted from ref. [4]).

The largest series of patients analyzing the etiology of HP was conducted in a Turkish population, involving 773 patients from multiple tertiary care institutions [4]. This study showed that the most common causes of HP were non-functioning PitNET (20.9%), Sheehan’s syndrome (13.8%), lactotroph PitNET (11.1%), and idiopathic causes (10.6%). The distribution of the PitNETs associated with HP is shown in Figure 2 [4].

It is important to consider the size and histologic type of pituitary tumors when evaluating the possibility of an associated HP. In this regard, it has been reported that the prevalence of HP in newly diagnosed adult patients with macroadenomas is 39.9% for non-functioning PitNETs, 27.3% for lactotroph PitNETs, and 14% for somatotropic PitNETs [16]. In addition, a study of adult men with lactotroph PitNETs showed that HP affected between 75.0% and 90.9% of patients with tumors of various sizes, and that larger tumors correlated with lower free thyroxine (FT4) levels after treatment [17].

HP is very common in patients with craniopharyngioma, with high rates observed preoperatively and potentially exacerbated postoperatively, affecting quality of life, morbidity, and mortality in these patients [18]. A retrospective study of 212 cases of craniopharyngioma patients undergoing total tumor removal surgery showed that 83.5% of patients had preoperative HP (36 of them panHP). Postoperatively, the condition of HP worsened in the early stages, with abnormal rates of 60.1% for the hypothalamic–pituitary–adrenal axis and 58.3% for the hypothalamic–pituitary–thyroid axis during the follow-up period [19].

Other neoplasms and tumor-like lesions of the sellar region associated with HP are pituitary carcinomas, pituicytomas, meningiomas, gliomas, gangliocytomas, metastases (especially lung and breast), Rathke’s pouch cysts, arachnoid membrane cysts, epithelial cysts (dermoid and epidermoid), hamartomas, germ cell tumors, and neurocytomas [20,21,22,23].

### 2.2. Pituitary Surgery

Pituitary surgery may result in HP due to the removal of normal peritumoral pituitary tissue. Its occurrence depends on several factors, including the following: (1) the size and location of the adenoma (higher risk in macroadenomas (≥10 mm) and those affecting critical areas of the pituitary or surrounding structures); (2) the presence of invasion and compression of normal pituitary tissue (higher risk in invasive PitNETs or those severely compressing normal pituitary tissue); (3) the type of surgery (transsphenoidal surgery, especially with the endoscopic technique, tends to be less invasive and has a lower risk of damaging normal pituitary tissue); (4) the skill and experience of the surgeon; and (5) the preoperative status of the pituitary gland (higher risk if the pituitary gland is already compromised by the adenoma) [24,25].

Damage to the pituitary stalk during surgery can lead to serious complications, mainly due to impaired pituitary hormone secretion resulting in HP with AVP deficiency and hyperprolactinemia.

### 2.3. Irradiation

Radiation used to treat PitNETs, head and neck tumors, intracranial neoplasms, or as adjuvant cranial irradiation for acute lymphoblastic leukemia can cause HP through several mechanisms, including (1) direct damage to pituitary cells; (2) disruption of the pituitary vasculature (damage to the microvasculature and vascular fibrosis that impairs adequate blood supply to the pituitary); (3) hypothalamic dysfunction (affecting the secretion of pituitary hormone-stimulating hormones); and (4) chronic and progressive processes of cellular and vascular damage [26].

The severity and frequency of radiation-induced hormone deficiencies correlate with the total dose of radiation delivered to the hypothalamic–pituitary region, resulting in irreversible and progressive deficiencies in anterior pituitary hormone secretion [27,28,29].

In children, doses above 18 Gy in the hypothalamic–pituitary region increase the risk of GH deficiency, doses between 18 and 30 Gy may result in precocious puberty as a consequence of activation of the gonadotropic axis, and doses above 30–40 Gy increase the risk of other hormone deficiencies. In adults, doses above 30 Gy, and especially those above 50 Gy, to the pituitary gland require close monitoring of the pituitary function [30,31,32].

HP occurs after both fractionated and single-dose radiation therapy. Fractionated radiation therapy is associated with ACTH, TSH, and Gn deficiency in approximately 20% of patients at 5 years and up to 30% at 10 years [33,34]. Stereotactic radiosurgery for recurrent or residual non-functioning PitNETs has been associated with preservation rates of pituitary function at 5, 8, 10, and 15 years of 83%, 81%, 78%, and 71%, respectively [35].

The prevalence of HP following cranial irradiation of the hypothalamus area varies depending on the population studied and the radiation doses received [36]. The prevalence of radiation-induced HP in adults with gliomas distant to the hypothalamic–pituitary area can be as high as 84.5%, with GH deficiency being the most common, affecting 82.8% of patients [32,37]. In addition, irradiated patients with skull base meningiomas have at least one hormone deficiency in 38% and complete HP in 13% [38].

### 2.4. Pituitary Apoplexy

Pituitary apoplexy is a critical condition characterized by hemorrhage or infarction of the pituitary gland, often caused by a pituitary adenoma [39,40]. It can present with symptoms such as headache, visual disturbances, and hormonal deficiencies, requiring urgent attention and sometimes urgent surgical decompression. Pituitary apoplexy may result in acute hormone deficiency due to hemorrhagic necrosis of the pituitary tissue. This may result in HP due to impaired hormone production by adenohypophyseal cells. In particular, involvement of the corticotropic axis may result in decreased cortisol levels, leading to symptoms such as fatigue, weakness, hypotension, and impaired level of consciousness.

The clinical course of HP in pituitary apoplexy is variable and depends on several factors, including tumor size and histologic type, hemorrhage size and degree of pituitary compression, preoperative adenohypophyseal function, and development of local surgical complications [41,42]. In some cases, patients may experience partial or complete recovery of pituitary function after treatment; however, recovery after surgery does not always occur. In many cases, persistent HP develops. Gn deficiency is the most frequent deficit, followed by ACTH and TSH deficiency. GH deficiency is also frequent, although it is not always assessed at the time of diagnosis.

It has been suggested that serum PRL might serve as a predictor of the recovery of pituitary function after pituitary apoplexy [43]. The presence of hypoprolactinemia at the time of diagnosis of pituitary apoplexy would indicate a greater degree of destruction of the pituitary parenchyma and, therefore, a lower probability of recovery of pituitary hormone function [43]. A recent meta-analysis found no statistically significant difference in recovery from HP between conservative treatment and surgery in a total of 908 patients with pituitary apoplexy in 15 studies. Conservative treatment was shown to be superior to early surgery for complete pituitary recovery [44].

Sheehan’s syndrome, or postpartum HP, is a medical condition that results from necrosis of the pituitary gland following postpartum hemorrhage [45]. The prevalence of Sheehan’s syndrome may vary by country. For example, in nations such as India, its incidence is relatively high due to high rates of postpartum hemorrhage. However, in developed countries, it is increasingly uncommon, which is attributed to advances in obstetric care and management of postpartum hemorrhage [46,47]. This syndrome may be associated with single or multiple pituitary hormone deficiencies, with clinical manifestations including secondary amenorrhea (central hypogonadism, CeHg), lactation failure (PRL deficiency), central hypothyroidism, CeH (TSH deficiency), secondary adrenal insufficiency (ACTH deficiency), and GH deficiency, with posterior pituitary involvement being less common [47,48].

### 2.5. Autoimmune Hypopituitarism

Autoimmune HP-associated diseases include lymphocytic hypophysitis, immune checkpoint inhibitor (ICI)-induced hypophysitis, IgG4-related hypophysitis, and paraneoplastic autoimmune hypophysitis (anti-PIT-1 hypophysitis and isolated autoimmune paraneoplastic ACTH deficiency) [49,50,51,52,53,54,55].

The prevalence of HP in lymphocytic hypophysitis varies, with somatotropic, adrenal, gonadal, and thyroid axis insufficiency reported in 44.4%, 33.3%, 33.3%, and 27.8% of patients, respectively. Additionally, AVP deficiency is present in 44.4% of patients [56].

Immunotherapy-associated hypophysitis develops in up to 14% of patients treated with ipilimumab, a cytotoxic T-lymphocyte antigen-4 (CTLA-4) inhibitor [57,58,59]. This type of hypophysitis is more frequent in men, generally over 60 years of age, as opposed to what occurs in primary lymphocytic hypophysitis, which generally develops in women (female–male ratio 5:1) with a mean age of 35 years and often related to the gestational or postpartum period [58]. Pituitary function is impaired in all patients. CeH and CeHg occur in 100% of cases, followed by hypoprolactinemia (92%), secondary adrenal insufficiency (50%), and finally, GH deficiency (17%). Both AVP deficiency [60] and syndrome of inappropriate antidiuretic hormone secretion (SIADH) [61] associated with ipilimumab treatment are rare. On the other hand, hypophysitis associated with immunotherapy using anti-programmed death-1 (PD-1) monoclonal antibodies, such as nivolumab and pembrolizumab, or antibodies directed against programmed cell death-ligand 1 (PD-L1), such as atezolizumab and durvalumab, is much less frequent (up to 0.5%) [62]. Secondary adrenal insufficiency due to isolated ACTH deficiency associated with ICI has been characterized recently [51]. Since its first description in 2016 [63] to the present, fewer than 200 patients have been reported [51]. This disease is generally associated with the use of anti-PD-1 monoclonal antibodies, mainly nivolumab, responsible for 60% of cases.

IgG4-related hypophysitis is a rare disease with a prevalence of 4% of all cases of HP and 30% of all cases of hypophysitis [64]. The mean age at diagnosis is 64 and 67 years in men and women, respectively, with a male–female ratio of 2.4:1. Clinically, it presents as panHP, anterior HP, and AVP deficiency in 52.4%, 26.2%, and 17.9% of patients, respectively.

Recently, a new clinical entity of paraneoplastic autoimmune hypophysitis has been proposed [54,55]. In this case, ectopic expression of pituitary antigens present in tumors causes a breakdown of immune tolerance, resulting in the production of autoantibodies and autoreactive cytotoxic T cells that specifically damage pituitary cells. Anti-PIT-1 hypophysitis is a form of paraneoplastic syndrome defined by (1) the presence of acquired GH, PRL, and TSH deficiency; (2) the detection of anti-PIT-1 antibodies or PIT-1 reactive T cells in the circulation; and (3) the coexistence of thymoma or malignancy [54,65]. On the other hand, ectopic ACTH expression in the tumor could induce autoimmunity against corticotropic cells and develop isolated autoimmune paraneoplastic ACTH deficiency in the form of paraneoplastic syndrome [54].

Autoimmune hypothalamitis has recently been considered as a new isolated autoimmune disease involving the hypothalamus. It is associated with HP, AVP deficiency, and the presence of anti-hypothalamic antibodies [66].

### 2.6. Empty Sella Turcica Syndrome

Empty sella turcica may be primary (idiopathic) or secondary, the latter being associated with a history of previous pituitary pathologies as well as surgical intervention, pharmacological treatments, or radiotherapy in the sella region. In most cases, the empty sella turcica is a neuroradiological finding without clinical implications. However, empty sella syndrome is defined by the presence of pituitary hormone dysfunction and/or neurological symptoms due to the possible coexistence of idiopathic intracranial hypertension [67]. The prevalence of HP associated with primary empty sella syndrome varies from 31% to 65% [68,69].

### 2.7. Head Trauma

Post-traumatic HP is a well-recognized clinical consequence of TBI [70]. Its development depends on the severity of the TBI, and it has been associated with several hormonal deficiencies, including GH (9–36%), TSH (2–33%), ACTH (10%, increasing to 50% after severe TBI), and AVP (15–51%) [70,71]. The pituitary stalk is also vulnerable to head trauma.

### 2.8. Infiltrative Diseases

Systemic infiltrative diseases are a group of relatively rare diseases that involve the deposition of substances or cells in multiple organs, including the endocrine glands. Examples of such diseases include inherited hemochromatosis, systemic amyloidosis, sarcoidosis, and Langerhans cell histiocytosis [72].

Iron accumulation in the pituitary gland due to hemochromatosis can lead to pituitary dysfunction [73,74,75]. The most common associated hormonal disorder is CeHg due to selective iron deposition in the gonadotropic cells [73]. Sexual function and sex hormone concentrations may normalize after iron depletion treatment with repeated phlebotomies [76,77].

While pituitary function is generally maintained in patients with systemic amyloidosis, isolated cases of HP associated with this condition have been documented [78,79,80].

It is uncommon for sarcoidosis to affect the pituitary gland. In fact, central nervous system involvement due to sarcoidosis (neurosarcoidosis) develops in only 5% of sarcoidosis patients, while hypothalamic–pituitary sarcoidosis occurs in only 9–18% of neurosarcoidosis cases [81]. The main hormonal alterations are Gn deficiency, AVP deficiency, hyperprolactinemia, and CeH. It is possible for HP to persist or progress despite improvement in imaging of lesions involving the hypothalamus, infundibulum, or pituitary after immunomodulatory therapy [81,82].

Involvement of the anterior pituitary has been observed in 5–20% of cases in Langerhans cell histiocytosis [83,84,85]. The most frequent hormone deficiency after AVP is GH [86]. Secondary hypogonadism has also been documented [87].

The hypothalamus can be affected by several infiltrative diseases, such as sarcoidosis [88] and Langerhans cell histiocytosis [72,83,84,88,89]. While hypogonadotropic hypogonadism is the most common hormonal disorder in sarcoidosis (present in 89% of cases) [82], AVP deficiency is the most common in Langerhans cell histiocytosis, affecting 15–50% of cases [84].

### 2.9. Infections

Infections can be a cause of HP, especially in immunosuppressed populations or in endemic areas [90]. They can affect the pituitary gland in various ways, causing inflammation or necrosis, which can compromise pituitary hormone production. Various causes have been described: bacteria (tuberculosis, syphilis, and leptospirosis), viruses (SARS-CoV-2 and HIV), fungi (Candida and Aspergillus), and parasites (*Toxoplasma gondii*) [90].

Pituitary abscess is an uncommon but serious condition accounting for less than 1% of pituitary lesions and characterized by an infectious process resulting in the accumulation of purulent material within the sella turcica. The most frequently associated bacterial and fungal microorganisms are Staphylococcus aureus (7.8%) and Aspergillus (8.8%), respectively. Anterior HP develops in 41.1% of patients and AVP deficiency in 24.8% [91].

A variety of microorganisms can affect the hypothalamus. These include bacteria, viruses, fungi, and parasites. Routes of infection may include direct spread from adjacent anatomical sites, previous central nervous system infections, and hematogenous spread. The most common risk factors are preexisting hypothalamic–pituitary lesions, sellar and suprasellar surgery, meningitis, sinusitis, and immunocompromised states [91].

### 2.10. Drugs

Pharmacologic HP refers to a decrease in pituitary function caused by the use of certain medications. This type of HP can occur when drugs interfere with the production or release of pituitary or hypothalamic hormones.

Opioid suppression of the hypothalamic–pituitary axis may result in hypogonadotropic hypogonadism, central adrenal insufficiency, and hyperprolactinemia [92].

Prolonged use of glucocorticoids at supraphysiologic doses prescribed as anti-inflammatory and immunosuppressive agents can suppress the function of several hypothalamic–pituitary axes, resulting in a decrease in the production of pituitary hormones such as ACTH, TSH, and Gn, which may become clinically evident during steroid treatment (CeH and/or CeHg) or after withdrawal (secondary adrenal insufficiency) [93].

In addition to glucocorticoids, other drugs can cause central hypothyroidism. These include recombinant human GH (rhGH) therapy, somatostatin analogs, selective RXR ligands such as bexarotene, salicylates, certain antiepileptic drugs (carbamazepine, oxcarbazepine, and valproic acid), and drugs that interfere with the neurodopaminergic system such as dopamine or its agonists [94,95]. Addictions associated with CeH include glue sniffing [96] and morphine [97].

Finally, several drugs that are accompanied by hyperprolactinemia, such as antipsychotics, neuroleptic-like medications, antidepressants, histamine receptor type 2 antagonists, proton pump inhibitors, some antihypertensive agents, and estrogens may result in hypogonadotropic hypogonadism [92,98,99].

### 2.11. Toxins

HP is the most common endocrine manifestation of snakebite envenomation, primarily from the bite of Russell’s viper (*Daboia russelii* and *D. siamensis*), and may develop at either an early or late stage [100,101].

### 2.12. Functional Hypopituitarism

Functional HP refers to a decrease in pituitary hormone production that is not due to a structural lesion of the gland but to systemic or functional factors. These include acute or chronic systemic diseases, anorexia nervosa, obesity, intense and prolonged exercise, acute stressful situations such as physical trauma or major surgery, intracranial hypertension without an identifiable mass (pseudotumor cerebri), among others.

### 2.13. Congenital Diseases

Defects in the embryonic development of the pituitary gland can result in HP. These defects include various congenital anomalies such as pituitary agenesis and hypoplasia and the septo-optic dysplasia syndrome (de Morsier’s syndrome) characterized by the classic triad of optic nerve hypoplasia, hypoplasia of the hypothalamic–pituitary axis associated with HP (62–80%, preferably GH deficiency), and midline brain defects such as agenesis of the septum pellucidum (60%) and/or corpus callosum [102]. Another clinical entity associated with HP is pituitary stalk disruption syndrome, which is characterized by the triad of thin/absent pituitary stalk, ectopic posterior pituitary, and anterior pituitary aplasia/hypoplasia [103].

Several hormone mutations (GH1, bio-inactive GH, FSHβ, LHβ, TSHβ, POMC), transcription factor defects (PITX2, TBX19, DAX1, NR5A1, NR0B), and hormone receptor mutations (GHRH, CRH, GnRH, TRH receptor mutations) can result in isolated hormone deficiencies. There are also mutations associated with multiple pituitary hormone deficiencies, such as defects in transcription factors (mutations in HESX1, SOX 2/3, LHX3/4, PROP1, POU1F1 or PIT1, IGSF1), and in the enzyme prohormone convertase (PC1) [104,105,106,107].

A number of genetic hypothalamic disorders are associated with HP due to abnormalities in the development and function of the hypothalamus and, consequently, in the production of anterior pituitary hormones [108]. These include Kallmann syndrome (hypogonadotropic hypogonadism) [109], septo-optic dysplasia (HP) [110], Prader–Willi syndrome (hypogonadotropic hypogonadism and GH deficiency) [111], and Bardet–Biedl syndrome (hypogonadotropic hypogonadism) [112].

## 3. Update and Advances in the Clinical Diagnosis of Hypopituitarism

Evaluation of pituitary hormone function should be performed in all patients with symptoms or suspicion of HP, in those at risk of HP (radiation therapy, cranio-encephalic trauma, brain injury, etc.), or in those with known hypothalamic–pituitary disease. In the initial evaluation of a patient with suspected HP, the anterior pituitary hormones and their peripheral hormones (ACTH, cortisol, TSH and free T4, FSH, LH, testosterone in men and estradiol in women, PRL, GH, and insulin-like growth factor-1 (IGF-1) should be assessed. Although baseline hormone determinations are usually sufficient to assess the thyroid, gonadal, and lactotropic axes, functional tests of pituitary reserve stimulation are often necessary to adequately assess the corticotropic and somatotropic axes [88]. The main clinical manifestations and diagnostic approaches to hormone deficiencies in HP are summarized in Table 2.

### 3.1. ACTH Deficiency

ACTH deficiency is accompanied by cortisol deficiency (secondary adrenal insufficiency) [113]. Its clinical presentation can be acute or chronic. In the acute form, accompanying symptoms include weakness, asthenia, dizziness, nausea, vomiting, hypotension, low level of consciousness, and even circulatory shock. In the chronic form, the associated symptoms and signs are asthenia, anorexia, nausea, vomiting, headache, myalgia, abdominal pain, decreased ability to concentrate, weight loss, skin pallor, and hypoglycemia [113,114,115].

ACTH deficiency usually occurs in association with other pituitary hormone deficiencies and rarely as an isolated hormone deficiency [113]. ACTH deficiency combined with LH further exacerbates the body hair loss associated with hypogonadism, especially in women, due to reduced adrenal androgen production. Hyponatremia is common in secondary adrenal insufficiency due to reduced renal free water clearance resulting from cortisol deficiency, which may be enhanced when associated with CeH. In contrast to primary adrenal insufficiency, hyperkalemia does not occur. This is because the function of the renin–angiotensin–aldosterone system (RAAS) remains intact.

The presence of a basal serum cortisol level (8:00–9:00 a.m.) < 3 μg/dL, confirmed by a second measurement, together with a normal or low plasma ACTH concentration, is highly suggestive of secondary adrenal insufficiency. In contrast, a non-stress basal cortisol level > 15 μg/dL virtually excludes this diagnosis. Patients with cortisol levels between 3 and 15 μg/dL require dynamic testing to confirm or rule out adrenal insufficiency [116]. It is recommended to perform serum cortisol measurement at least 18–24 h after the last dose of hydrocortisone to avoid interference with the results.

The insulin tolerance test (ITT) is the most sensitive test for assessing pituitary ACTH reserve. In this test, intravenous insulin (0.05–0.15 IU/kg) is administered, resulting in hypoglycemia (blood glucose < 40 mg/dL, 2.2 mmol/L), usually after 20–30 min. This is a potent stimulus for ACTH and GH secretion. The levels of glucose and cortisol are measured at −30, 0, 30, 60, and 120 min. A normal response is considered to be a cortisol peak > 18 μg/dL (500 nmol/L). However, this test is not without risk and is contraindicated in the elderly and in patients with a history of ischemic heart disease and seizures. Therefore, it is usually replaced by the rapid ACTH stimulation test, which minimizes the risks and can assess the adrenal reserve and, indirectly, the corticotropic function of the pituitary gland. In this case, exogenous ACTH (250 μg ACTH 1–24 synthetic ACTH) is administered intravenously, followed by the determination of serum cortisol at 0, 30, and 60 min. A cortisol response of ≥18 μg/dL (500 nmol/L) at 30 to 60 min is indicative of normal adrenal reserve [88,116,117]. Because ACTH is a trophic hormone that is essential for maintaining the integrity of adrenal function, this test is appropriate only when performed approximately 2–4 weeks after the presumed onset of ACTH deficiency.

### 3.2. TSH Deficiency

TSH deficiency is associated with a deficiency in thyroid hormone synthesis (secondary hypothyroidism). Clinical manifestations are usually similar but milder than those of primary hypothyroidism. It presents with delayed growth and short stature in children. In adults, it is associated with fatigue, muscle weakness, depression, cognitive decline, cold intolerance, constipation, weight gain, pale and dry skin, hair loss, bradycardia, slowed reflexes of relaxation, and menstrual disorders (oligomenorrhea and amenorrhea). In combined deficiency with ACTH, hyponatremia is more common, and weight gain is less frequent. Combined deficiency with Gn is associated with normocytic and normochromic anemia [104,118].

The diagnosis of central or secondary hypothyroidism is made by evaluating serum free T4 and TSH levels. A free T4 level below the laboratory reference range together with a low, normal, or slightly elevated TSH (<10 mcU/mL) in the context of pituitary disease usually confirms the diagnosis. With the current use of ultrasensitive methods for TSH quantification, stimulation testing has become unnecessary [116,118,119].

### 3.3. Gonadotropin Deficiency

Gn deficiency is associated with a deficiency in the production of the gonadal sex steroids (testosterone in men and estradiol in women) responsible for sexual development, maintenance of secondary sexual characteristics, and fertility (hypogonadotropic hypogonadism or secondary hypogonadism) [88,120].

#### 3.3.1. Men

Lack of gonadal stimulation during fetal life results in suboptimal genital development, resulting in reduced testicular size and external genital abnormalities [121]. The phenotype of Gn deficiency in the male neonate includes features such as the absence of adequate testicular development and the presence of a micropenis at birth. These neonates may also present with cryptorchidism or high-positioned testes.

In male adolescents, it manifests as delayed puberty (absence of testicular enlargement before 14 years of age). In the adult male, testosterone deficiency is associated with erectile dysfunction; decreased libido; decreased muscle mass, strength, and bone mass and erythropoiesis; and reduced spermatogenesis with impaired fertility, hair growth, and testicular atrophy [120,122,123].

The diagnosis of hypogonadotropic hypogonadism in the male adult is made by an initial clinical evaluation for symptoms and signs of hypogonadism and determination of serum levels of total testosterone, LH, and FSH on at least two different days, in the absence of acute/subacute illness and preferably before 10 a.m., after an overnight fast combined with serum PRL. In hypogonadotropic hypogonadism, low testosterone levels are expected along with low or normal levels of LH and FSH [88,116,120,122].

Only 2% of circulating testosterone is free, while 54% is bound to albumin and 44% is bound to sex-hormone-binding globulin (SHBG). Therefore, low borderline total testosterone levels may be associated with conditions or medications that affect SHBG and albumin. While aging, hyperthyroidism, liver disease, HIV, elevated estrogen concentrations, and antiepileptic drugs are associated with increased SHBG, obesity, insulin resistance, type 2 diabetes, hypothyroidism, excess growth hormone, exogenous androgens/anabolics, glucocorticoids, progestogens, and nephrotic syndrome are associated with decreased SHBG. In these cases, it may be advisable to measure bioavailable testosterone (http://www.pctag.uk/testosterone-calculator/, accessed on 10 September 2024) [120,124].

Finally, genetic testing can be used for the identification of variants associated with hypogonadotropic hypogonadism, particularly in cases of congenital hypogonadism [13,108].

#### 3.3.2. Women

In female adolescents, hypogonadotropic hypogonadism manifests as delayed puberty (absence of breast development before age 13 and the absence of menstruation, primary amenorrhea). In adult women, estradiol deficiency is associated with menstrual disorders (oligomenorrhea or secondary amenorrhea), infertility, loss of libido, dyspareunia, fine wrinkles, breast atrophy, and decreased secondary sexual hair, especially when combined with ACTH deficiency, premature atherosclerosis, and predisposition to osteoporosis [120,122].

The diagnosis of hypogonadotropic hypogonadism in women is based on clinical suspicion, preferably the presence of oligomenorrhea or secondary amenorrhea along with other symptoms related to estrogen deficiency such as hot flashes, vaginal dryness, and decreased libido. Measurement of the serum estradiol, FSH, and LH concentrations is recommended. In hypogonadotropic hypogonadism, estradiol levels are decreased, and FSH and LH levels are low or in the normal range. Other causes of menstrual abnormalities and ovarian dysfunction, such as hyperprolactinemia, thyroid dysfunction, and hyperandrogenism, should always be excluded. In postmenopausal women, the absence of elevated FSH and LH levels is sufficient to diagnose gonadotropic dysfunction, provided the patient is not on hormone replacement therapy. It is recommended that dynamic GnRH testing not be performed as it does not provide additional useful diagnostic information compared with baseline Gn levels [116]. Genetic testing can be performed to identify congenital causes of hypogonadotropic hypogonadism [13,108].

### 3.4. Growth Hormone Deficiency

Growth hormone deficiency (GHD) is a syndrome caused by the insufficient production of GH by the pituitary gland. This deficiency can occur in children, resulting in abnormally slow growth, short stature, and other problems related to physical and metabolic development. In adults, it manifests as changes in body composition such as decreased muscle mass, increased body fat, decreased bone density, and changes in metabolism. Clinically, it can manifest as reduced health-related quality of life; fatigue; reduced exercise tolerance; obesity; anxiety, depression and social isolation; bone fractures; and an increased risk of cardiovascular disease [125,126,127,128,129,130].

GHD should be suspected in any patient with clinical signs or risk of developing HP, as well as in those who received rhGH treatment during childhood and adolescence. The diagnosis of GHD should include clinical evaluation, serum IGF-1 quantification, and GH stimulation testing [131].

In children, growth retardation and short stature should be the primary concerns, while in adults, symptoms such as fatigue, depression, increased body fat, decreased muscle mass, and decreased quality of life should be considered.

Measurement of serum IGF-1 levels is an important initial test because its levels are relatively stable and reflect GH activity. However, because normal IGF-1 levels do not always rule out GHD (20% of adults with GHD may have normal IGF-1 levels, especially in men) [132], and low levels are not always associated with GHD, GH stimulation testing is almost always necessary. In individuals presenting with HP affecting three or more hormonal axes, the presence of low IGF-1 levels may be sufficient to diagnose GH deficiency without the need for stimulation testing [88,116].

Because serum GH levels can fluctuate throughout the day, stimulation testing is recommended to confirm deficiency. ITT is the gold standard test for assessing GHD. Intravenous insulin (0.05–0.15 U/kg) is administered to induce hypoglycemia (blood glucose < 40 mg/dL or 2.2 mmol/L), and serum GH is determined at various times: 0, 30, 60, and 120 min. A GH response < 3 ng/mL is indicative of GHD. In the glucagon test, glucagon is administered (1 mg intramuscular; 1.5 mg if weight is > 90 kg), and GH is measured at various time points: 0, 30, 60, 90, 120, 150, 180, 210, and 240 min. A GH value < 3 µg/L suggests GHD. In the GHRH + arginine test, GHRH (1 µg/kg intravenous, maximum 100 µg) is administered followed by an infusion of arginine (0.5 g/kg, maximum 35 g) for 30 min. GH is measured at various points (0, 30, 45, 60, 75, 90, 105, 120 min). A GH value < 4 ng/mL indicates GHD [116,132,133,134].

### 3.5. Prolactin Deficiency

PRL deficiency is usually seen in association with other anterior pituitary hormone deficiencies. The main cause is treatment with dopaminergic agonists. Other etiologies include Sheehan syndrome, pituitary apoplexy, radiation therapy, TBI, and pituitary adenomas [98].

Hypoprolactinemia is associated with the inability to lactate, also known as agalactia. Recent studies have shown that hypoprolactinemia may have an influence beyond lactation, such as an increased risk of metabolic abnormalities, including obesity, abnormal lipid profile, insulin resistance, type 2 diabetes, and fatty liver, as well as fertility problems and sexual dysfunction [98,135]. Hypoprolactinemia should be considered in the clinical evaluation of patients at risk for HP, especially those with pituitary apoplexy [42] and those who have undergone radiotherapy [135].

The diagnosis of hypoprolactinemia is not clearly defined and may vary depending on the criteria used. In general, hypoprolactinemia is considered when serum PRL levels are <5 ng/mL in men and <7 ng/mL in women, or when peak PRL after TRH stimulation test is <18 ng/mL in men and <41 ng/mL in women [98].

### 3.6. Arginine Vasopressin Deficiency

AVP deficiency, also known as central diabetes insipidus, is characterized by a variety of symptoms resulting from the body’s inability to concentrate urine and retain water at the renal level. The main symptoms are hypotonic polyuria (urine volume > 3 L per day and urine osmolarity < 300 mOsm/kg) and polydipsia (>3 L per day) to compensate for dehydration. If fluid intake does not adequately compensate for water loss, patients may suffer from dehydration, which is manifested by symptoms such as dry skin and mucous membranes, fatigue, weakness, irritability, confusion, and in severe cases, disorientation, seizures, or coma [88,136,137].

If AVP deficiency is suspected, it is recommended to measure natremia and plasma osmolality. If both parameters are normal, a water deprivation test (at least 16 h) can be performed to indirectly measure AVP activity by analyzing the ability to concentrate urine. This test consists of suppressing the intake of water and all fluids to produce dehydration and provoke a potent stimulus for AVP secretion.

If urine osmolality remains below 300 mOsm/kg after the water deprivation test, there is most likely a complete loss of AVP release or action. After the water deprivation test, a desmopressin (DDAVP) test is performed by administering 1 μg DDAVP acetate subcutaneously or 10 μg intranasally. In cases of complete AVP deficiency, urine osmolality increases by ≥50%, whereas in cases of AVP resistance, the increase is less than 50%.

If after the water deprivation test the urinary osmolality remains between 300 and 800 mOsm/kg, the cause of polyuria may be partial AVP deficiency, partial AVP resistance, or primary polydipsia. In partial AVP deficiency, there is a 9–50% increase in urinary osmolality one hour after DDAVP administration, whereas in partial AVP resistance or primary polydipsia, the increase is <9%.

Another way to assess the presence of AVP deficiency is to analyze unstimulated plasma levels of copeptin, an indirect biomarker of AVP secretion that is easily measured. The presence of copeptin levels > 21.4 pmol/L indicates AVP resistance. If plasma copeptin is ≤21.4 pmol/L, it is recommended that the copeptin stimulation test be performed with hypertonic saline unless contraindicated. Alternatively, the copeptin stimulation test can be performed with intravenous arginine. The diagnosis of AVP deficiency is established when the post-stimulation copeptin level is ≤4.9 pmol/L after administration of hypertonic saline or ≤3 pmol/L after administration of arginine.

Lastly, the presence of hypernatremia (Na > 147 mmol/L) associated with basal copeptin levels ≤ 4.9 pmol/L is indicative of AVP deficiency, whereas unstimulated copeptin levels ≥ 21.4 pmol/L would indicate AVP resistance [136,137,138,139,140].

### 3.7. Oxytocin Deficiency

Oxytocin deficiency has been less studied than other pituitary hormone deficiencies, but its clinical importance is beginning to be recognized. Oxytocin plays a critical role in lactation by facilitating milk secretion; therefore, oxytocin deficiency can lead to breastfeeding difficulties. Oxytocin is also involved in the regulation of social interactions and affective behavior. Its deficiency may be associated with problems in emotional bonding and interpersonal relationship formation. Finally, oxytocin has anxiolytic effects and may help reduce stress. A deficiency of this hormone may contribute to increased anxiety and a more intense stress response [141,142,143,144,145,146,147].

Biochemical tests used to diagnose oxytocin deficiency typically involve measurement of its plasma concentration, although this can be challenging due to the rapid degradation of the hormone and its fluctuating levels in the circulation [147]. Recent studies have highlighted the importance of standardizing assay protocols to improve diagnostic accuracy, as variations in assay methods can lead to inconsistent results [147,148]. Therefore, further research is needed to refine diagnostic criteria and improve the reliability of oxytocin measurement techniques.

## 4. Pitfalls in the Diagnosis of Hypopituitarism

**Figure 3:**
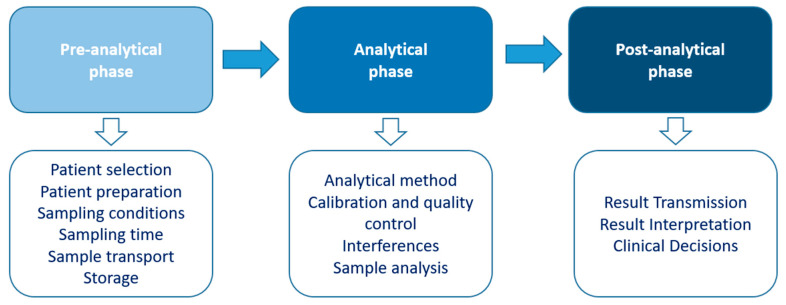
Pre-analytical, analytical, and post-analytical phases in hormonal evaluation.

The diagnosis of HP is mainly based on laboratory tests, although these tests have challenges and limitations that need to be properly understood and addressed, making a multidisciplinary approach essential. Three phases can be considered in the diagnostic process: pre-analytical, analytical, and post-analytical (Figure 3) [8].

In the pre-analytical phase, biological and technical factors that may affect hormone measurement, such as age, sex, menstrual cycle, time of day of sampling (hormonal circadian rhythms), stress during blood sampling, presence of hemolysis, proper sample preparation and storage, and sample transport conditions, should be known.

It is important to understand the circadian rhythms of the various hormones in order to properly interpret hormone concentrations at different times of the day. ACTH and cortisol show a pronounced diurnal rhythm, with cortisol levels peaking in the early morning hours and gradually decreasing until midday, followed by a slight increase in the afternoon and a decline to their lowest levels around midnight. PRL follows a similar pattern to cortisol, with no significant increase in the afternoon without specific stimulation. GH is secreted in pulses and is significantly affected by the sleep–wake cycle, with the greatest amplitude of secretion during the first hours of sleep. Changes in the circadian rhythm, such as those experienced by shift workers, may affect this secretion pattern and result in lower nocturnal pulse amplitudes. TSH rises in the late afternoon, peaks in the morning, and declines throughout the day, therefore TSH determinations should ideally be performed after a normal night’s sleep. FT3 and FT4 have a less pronounced circadian pattern. Testosterone peaks in the morning, declines during the day, and rises again in the evening [8,149,150]. Acute stress is accompanied by increased secretion of pituitary hormones such as ACTH (and subsequently cortisol), GH, prolactin, and TSH [151,152]. Therefore, stress should be avoided during blood sampling to obtain true results of hormone determinations. It is important to know the patient’s current and past medications, because various medications can affect pituitary hormone concentrations. Thyroid hormone, glucocorticoid, testosterone, and estrogen therapies can suppress the corresponding pituitary hormones. In addition, the use of over-the-counter medications and the abuse of illicit substances such as steroids and opioids, which can affect the gonadotropic and corticotropic axes, should be considered. In assessing the somatotropic axis, oral estrogen use may cause growth hormone resistance, while testosterone may increase GH secretion. The role of lithium on thyroid function must also be considered, as it can cause hyperthyroidism or hypothyroidism [152,153].

During the analytical phase, it is important to use appropriate methods and to consider biological variability when interpreting results. Quantification in clinical laboratories is mainly performed using immunoassays, while mass spectrometry is rarely used due to its complexity and cost. For the quantification of steroids, some laboratories have adopted liquid chromatography–mass spectrometry, which offers greater specificity and less interference. Although modern tests often provide information on the cross-reactivity of some substances (e.g., interference from the GH receptor antagonist pegvisomant in GH assays and from the aromatase inhibitor exemestane in estrogen assays), the possibility of cross-reactions can never be completely ruled out. Different antibodies can lead to interference in hormone determination, including heterophilic antibodies, human anti-animal antibodies, and autoantibodies. Biotin can cause both falsely low and high results in hormone determinations, underscoring the importance of being aware of its intake in patients and the need to perform additional tests or use alternative methods when interference is suspected [8,154].

Finally, in the post-analytical phase, it is essential to compare the results with appropriate reference intervals and to consider the biological variables such as age, sex, and other concomitant endocrine or metabolic diseases [8].

## 5. Update and Advances in the Treatment of Hypopituitarism

Hormone replacement therapy remains the mainstay of treatment for HP, although new therapeutic options are being investigated that may offer additional benefits to patients. Table 3 shows the main therapeutic interventions currently used in the treatment of hormone deficiency associated with HP.

### 5.1. Central Adrenal Insufficiency

Treatment of central adrenal insufficiency is based on hormone replacement with glucocorticoids, with hydrocortisone being the drug of choice. The recommended dose should approximate natural daily cortisol production, which ranges from 10 to 20 mg per day [155]. To mimic the circadian rhythm of cortisol, it is suggested that the total daily dose be divided into two or three doses, with the largest amount administered in the morning. In stressful situations (such as infection or surgery), the dose may be increased to 100 mg as an intravenous bolus, followed by a continuous infusion of 200 mg/day or frequent boluses of 50 mg every 6 h [155,156].

Hormone replacement therapy with glucocorticoids such as prednisone or dexamethasone is generally not recommended. This is due to their inability to simulate the circadian rhythm of cortisol, their considerable intra- and interindividual metabolic variability, and the high risk of overdose. However, prednisone could be considered in certain situations, such as in cases of poor adherence to hydrocortisone treatment due to the need for multiple daily doses or when hydrocortisone is not available [155]. The dose of prednisone used to treat secondary adrenal insufficiency may vary depending on the individual needs of the patient, the severity of the deficiency, and the response to treatment. The approximate prednisone equivalent dose for 20 mg of hydrocortisone is about 5 mg [88].

Patients with ACTH deficiency due to hypothalamic–pituitary disease or suppression of the hypothalamic–pituitary–adrenal axis after continuous exogenous glucocorticoid use do not require mineralocorticoid replacement because the RAAS is preserved in these cases [88,155].

Androgen deficiency may also occur in secondary adrenal insufficiency. Administration of dehydroepiandrosterone (DHEA) in doses of 10 to 25 mg per day may improve libido and emotional and mental well-being. However, due to the lack of sufficient data on its long-term safety, it is not recommended at this time [157,158].

Extended-release formulations of hydrocortisone have been developed to optimize the treatment of adrenal insufficiency by providing a more consistent and physiologic cortisol release profile throughout the day. These formulations allow for reduced variability in cortisol levels, improved quality of life and overall patient well-being, and potentially reduced side effects associated with fluctuations in cortisol levels [155]. Two modified-release (MR) hydrocortisone formulations have been developed: Plenadren^®^ and Chronocort^®^ [158]. Plenadren^®^ is a modified-release hydrocortisone with an immediate-release outer shell and a sustained-release core. Plenadren provides a longer serum cortisol profile compared with immediate-release hydrocortisone. It is available in 5 and 20 mg tablets, and the usual maintenance dose in adults is 20–30 mg/day. Chronocort^®^ uses multi-particulate technology, where an inert microcrystalline core is coated with a drug layer with polymeric layers that modulate drug release. The recommended dosing regimen is twice daily (10 mg at 7:00 a.m. and 20 mg at 11:00 p.m.), mimicking the circadian rhythm of cortisol.

### 5.2. Central Hypothyroidism

The treatment of CeH is mainly focused on the administration of hormone replacement therapy with levothyroxine (LT4) in monotherapy to normalize serum thyroid hormone levels, especially FT4 [88,118,119,159]. The combination of LT4 with LT3 has not shown superior efficacy to treatment with LT4, but it could be considered in some patients who continue to have symptoms despite treatment with LT4 [119].

The treatment of CeH should be initiated only after confirmation of the preservation of cortisol secretion or assurance that the patient is receiving adequate hydrocortisone replacement. In the case of coexisting central adrenal insufficiency, thyroid hormone replacement should be started after the initiation of steroid therapy to prevent adrenal crisis [160,161,162,163].

Serum TSH levels are not useful for monitoring treatment in CeH. Monitoring should be performed with FT4 levels, which should be in the upper half of the normal range [162,163].

In children and young adults, treatment can be started with a full replacement dose of LT4. In congenital CeH, it is essential to start high-dose LT4 as soon as possible (preferably within 2 weeks of birth) at doses similar to those used in primary congenital hypothyroidism (10–12 μg/kg body weight/day) to rapidly normalize FT4 concentrations and ensure optimal neurocognitive development. In milder forms of CeH, treatment may be initiated with lower doses of LT4 (5–10 μg/kg body weight/day) to avoid the risk of overtreatment [119].

For most adults with CeH, average daily doses of LT4, ranging from 1.2 to 1.6 μg/kg body weight/day (75–150 μg/day), are usually adequate. In elderly patients or those with longstanding hypothyroidism who are at risk for adverse effects, especially in the presence of ischemic heart disease or concomitant cardiovascular disease, LT4 treatment can be initiated with a lower daily dose (e.g., 25–50 μg of LT4), increasing the dose by 25–50 μg every 6–8 weeks until a dose of 1.0 to 1.2 μg/kg body weight/day is reached [119,162,163].

In pregnant women with CeH, a 25–50% increase in LT4 dose is recommended to ensure that FT4 concentrations remain in the upper quartile of the normal range [119].

### 5.3. Central Hypogonadism

Treatment of CeHg is based on hormone replacement therapy with sex steroids in both men (testosterone) and premenopausal women (estradiol and progesterone). If fertility is desired, treatment should be with GnRH or exogenous Gn.

#### 5.3.1. Men

Testosterone replacement therapy (TRT) is the standard treatment for CeHg in men. It improves the symptoms associated with hypogonadism (reduces fat mass; prevents anemia; and improves libido and sexual function, bone mineral density, energy levels, mood, muscle mass, and strength). However, it has limitations; for example, it is not recommended for men seeking fertility or those at high risk of polycythemia, thrombophilia, prostate cancer, or serious cardiovascular disease [124,164].

Daily testicular testosterone production in men ranges from 4 to 9 mg/day. Its secretion pattern is not constant, as it follows a circadian rhythm with a higher peak in the early morning hours (06:00–08:00 a.m.), gradually decreasing throughout the day (nadir at 08:00–09:00 p.m.), and then increasing again at night and during sleep to reach a new morning peak. It is important to consider this pattern of secretion when measuring blood testosterone, as levels can vary significantly depending on the time of day the sample is taken [165,166].

There are several options for testosterone administration, including intramuscular injections, transdermal patches, topical gels, or subcutaneous implants. The choice of method depends on patient preference and availability [88,116,167,168,169].

Long-acting testosterone injections, by administration of testosterone esters such as testosterone cypionate and testosterone propionate (100 mg and 250 mg ampoules for intramuscular administration) are generally administered every 2–4 weeks, with an interval between doses that may be reduced to 10 days depending on clinical and analytical response. These preparations cause fluctuations in serum testosterone levels (supraphysiological after administration and subtherapeutic before the next injection). To evaluate the response, an analytical hormonal control is performed in the mean between one dose and the following one.

The ultra-long-acting intramuscular injection is the intramuscular formulation of another testosterone ester, testosterone undecanoate. This is the formulation with the longest half-life (administered every 10–14 weeks), and it provides the most stable plasma testosterone levels. To evaluate the response, analytical determination should be performed at the end of the interval between doses, just before the next injection.

Testosterone preparations are also available for topical/transdermal administration. These include testosterone gels (1%, 1.62%, and 2% testosterone) and solutions (2% testosterone). A 5 g container of 1% testosterone gel contains 50 mg of testosterone. Since the absorption of testosterone in gel form is approximately 10% to 15% of the applied dose, this means that from a dose of 50 mg/day of testosterone gel, approximately 5 mg to 7.5 mg per day will be absorbed. These preparations achieve normal, relatively stable serum testosterone concentrations. In order to adjust the dosage of testosterone, it is advisable to measure the serum testosterone concentration in the morning prior to the application of testosterone from the third day after the start of treatment [164,167,168,169,170]. PSA levels, hematocrit, and bone mineral density should be monitored during follow-up.

Induction of fertility in men with CeHg due to Gn deficiency caused by organic pituitary lesions, GnRH deficiency, or GnRH resistance requires hormone replacement therapy with exogenous Gn, which stimulates sperm production and maintains testosterone levels. The main Gn used are human chorionic Gn (hCG) and FSH.

hCG is used to mimic the action of LH by stimulating Leydig cells in the testes to produce testosterone. FSH, on the other hand, stimulates spermatogenesis and can be administered from three main sources: (1) highly purified human menopausal Gn (hMG), which is extracted from the urine of postmenopausal women and contains mainly FSH activity, but also a small amount of LH; (2) highly purified urinary FSH (uFSH); and (3) recombinant FSH (rFSH), which is produced by genetic engineering and is pure, without LH. Because it is recombinant, it has a more consistent composition and may be preferred for its purity and potency [171,172].

Treatment begins with subcutaneous or intramuscular injection of hCG (1000–2000 IU, 3 times per week), with the dose adjusted according to serum testosterone levels and testicular growth. In some patients, hCG alone may be sufficient [173]. When normal, stable testosterone levels (400 and 800 ng/dL) are achieved (usually after 6 months of treatment), FSH is added (75 U, 3 times per week). If testicular size and sperm count remain inadequate (sperm count < 5 million/mL), the dose of FSH may be gradually increased to 150 U, 3 times per week [174]. Maintaining this regimen for 12–24 months usually induces testicular growth in most patients, spermatogenesis in 80%, and pregnancy rates around 50% [171].

Gn therapy, specifically the use of FSH to stimulate Sertoli cell proliferation prior to hCG administration, is a more recent and effective approach to improve spermatogenesis [171].

Men with central CeHg due to hypothalamic disease (GnRH deficiency) can be treated with hormone replacement therapy by pulsatile administration of GnRH through a subcutaneous infusion pump. The most common starting dose is 25 ng/kg body weight every 120 min, titrated according to testosterone concentrations. This form of treatment has been associated with earlier spermatogenesis and larger testes than combination Gn therapy [175]. Recently, it has been reported that in some patients with previous poor response to one year of HCG/HMG treatment, switching to pulsatile GnRH treatment can induce spermatogenesis [176].

#### 5.3.2. Women

Hormone replacement therapy for women with CeHg focuses on restoring hormone levels and relieving associated symptoms. In premenopausal women (under age 45), the goal of hormone replacement therapy is to prevent symptoms of estrogen deficiency, such as hot flashes, vaginal dryness, decreased libido, and long-term complications such as osteoporosis. It also aims to reduce the risk of cardiovascular disease and mortality [88,116,173,177]. In premenopausal women who have not undergone hysterectomy, progestogens are used in combination with estrogens to prevent endometrial hyperplasia and endometrial cancer. In those who have undergone hysterectomy, estrogens alone may be used if there are no contraindications, especially regarding the risk of breast cancer or thrombosis [88]. In postmenopausal women, estrogen doses should be individualized to relieve vasomotor symptoms and those caused by vaginal atrophy [116,178].

There are several different patterns of hormone replacement therapy. These include the following: (1) the sequential cycle, in which estrogens are given continuously every day, while progesterone is added for 10 to 14 days each month to induce menstruation after withdrawal; and (2) the combined continuous cycle, in which estrogens and progestogens are given together every day without interruption [179].

The preferred type of estrogen is 17-β-estradiol because it provides physiologic replacement and allows measurement of serum levels, if needed. Estrogens can be administered in a variety of forms, such as transdermal patches, gels, oral tablets, or vaginal rings, with the dose adjusted according to individual needs and response to treatment [179]. Patches and gels are usually preferred because they provide a more consistent release of the drug, which helps to avoid hormonal fluctuations. Transdermal therapy is the first choice for patients with pituitary disease affecting multiple axes, as it avoids the effects of oral estrogen on other hormone-transporting proteins. Oral estradiol tablets are administered in doses of 1–2 mg daily, while transdermal patches release 50–100 µg of estradiol daily and are applied once or twice a week, and gels are applied daily. Vaginal rings, although less commonly used for systemic hormone therapy, release estradiol steadily over several months. Switching from oral to transdermal estrogen administration in women also receiving rhGH results in a significant increase in serum IGF-1 levels of approximately 30%. This increase in IGF-1 levels results in a significant reduction in the dose of rhGH required to achieve therapeutic goals [180].

Progestogens can be administered as oral tablets, transdermal patches, or progestin-releasing intrauterine devices (IUDs). The dose and route of administration are tailored to individual needs. Oral tablets may contain micronized progesterone in doses of 100–200 mg daily, usually administered for 10–14 days of the monthly cycle, or synthetic derivatives such as medroxyprogesterone acetate (10 mg/day for 10–14 days). Transdermal patches combine estrogen and progestin and provide continuous release. Levonorgestrel-releasing IUDs (52 mg), an effective extended-release option, release progestin continuously for several years [179].

Fertility induction in women with CeHg is mainly focused on ovulation induction. This can be achieved by pulsatile administration of GnRH or by ovarian stimulation with Gn. The goal is to achieve mono-ovulation to avoid multiple pregnancies [181].

HMG, uFSH, and rFSH preparations have been used successfully for controlled ovarian hyperstimulation. Meta-analyses suggest that rFSH is superior to the other Gn in assisted reproductive cycles [182,183,184,185]. Controlled ovarian stimulation with rFSH results in multiple follicular recruitment. rFSH is administered at doses of 75–150 U/day, and follicular growth is monitored with transvaginal ultrasound and estradiol levels. After follicular maturation, defined as the presence of three or more follicles larger than 18 mm, hCG is administered at doses of 5000–10,000 IU to induce ovulation. Then, approximately 36 h after the hCG injection, intercourse is scheduled or intrauterine insemination is performed. In some cases, progesterone is administered to support the luteal phase and improve implantation rates [173,177,178,179,180]. Assisted reproductive treatment in women with HP typically achieves pregnancy rates ranging from 47% to 100%. In patients who have achieved pregnancy, live birth rates have ranged from 61% to 100% [177]. It has been suggested that the addition of rhGH to Gn improves ovarian stimulation and pregnancy rates in poor responders undergoing ovulation induction and controlled ovarian stimulation for in vitro fertilization [186,187,188,189,190,191].

Pulsatile administration of GnRH is considered the ideal treatment, because it induces more physiological ovulatory cycles. By naturally stimulating Gn secretion, the resulting serum FSH and LH concentrations are maintained within the normal range, reducing the likelihood of multifollicular development and ovarian hyperstimulation [192]. It requires that gonadotropic function be intact; therefore, it would be appropriate only for use in women with GnRH deficiency.

The most physiologic dose for iv administration is 75 ng/kg with a pulse interval of 60 to 90 min to mimic the normal pulsatile release of GnRH [193]. During the early follicular phase, the frequency of administration is every 90 min, while during the mid and late follicular phases, it is reduced to every 60 min. After ovulation, confirmed by ultrasound monitoring, the frequency of administration is increased to every 90 min and further reduced to every 240 min during the luteal phase to favor FSH secretion over LH secretion. Alternatively, a constant infusion of 5 mg GnRH can be maintained throughout the menstrual cycle. It is also possible to maintain the corpus luteum after ovulation by administering 500 units of hCG on days 7, 10, and 13 after ovulation. Once pregnancy is achieved, pulsatile GnRH administration is discontinued. Ovulation rates of 90% and pregnancy rates of 80% or higher have been reported in women treated with pulsatile GnRH [192,193,194,195].

### 5.4. Growth Hormone Deficiency

Treatment with rhGH is used in both children and adults with GHD. This therapy has demonstrated benefits in many parameters, including stimulation of growth in children, thereby improving height prognosis, and improvement in quality of life, body composition, exercise tolerance, bone mineral density, and several cardiovascular risk factors [196,197].

rhGH is usually administered by subcutaneous injection. The recommended starting dose of rhGH in children with GHD is 0.16 to 0.24 mg/kg/week (22–35 μg/kg/day) divided into six to seven daily subcutaneous injections, with individualized dose adjustment according to serum IGF-1 levels [31,198]. The dose of rhGH required during the transition period from adolescence to adulthood may vary depending on individual factors such as gender and concurrent treatments. The usual starting dose in adults is 0.2 to 0.4 mg daily subcutaneously (0.1 to 0.2 mg daily in those over 60 years of age), with increases of 0.1 to 0.2 mg daily every 6 weeks until serum IGF-1 levels are in the upper part of the normal range [116,199,200].

Monitoring of rhGH treatment is performed both clinically and analytically, including assessment of blood glucose, HbA1c, and lipids, as well as the measurement of IGF-1 levels to ensure compliance. In children, IGF-1 levels should be adjusted to the upper percentiles of the normal population for their age and sex, as the goal of treatment is to achieve adequate growth and normalize height. In adults, IGF-1 levels should be in the middle to upper range of reference values for their age group. If IGF-1 levels are too high, it is necessary to reduce the dose of rhGH to avoid the side effects and complications associated with GH overdose. Once the dose is stabilized, follow-up can be done every six months [88,116].

There are several long-acting (weekly) rhGH (LAGH) formulations that have been developed to relieve patients and their families of the burden of daily injections, thereby improving treatment compliance. These include somapacitan (Sogroya^®^, Novo Nordisk; dose: children: 0.16 mg/kg/week; adults: 1.5 mg/week), a GH modified by the addition of an amino acid that provides increased affinity for binding to endogenous albumin; somatrogon (Ngenla^®^, Pfizer; dose: children: 0.66 mg/kg/week), a fusion protein containing the amino acid sequence of GH and three copies of the C-terminal peptide of hCG; and lonapegsomatropin (Skytrofa^®^, Ascendis; dose: children: 0.24 mg/kg/week), a reversibly pegylated GH. The efficacy, safety, and tolerability of these LAGHs appear to be similar to daily rhGH injections in both children and adults [200,201,202]. However, several issues remain to be addressed regarding the use of LAGH formulations, including the long-term efficacy, the immunogenicity of LAGH molecules, the optimization of therapeutic monitoring, and the potential long-term effects of a non-physiologic GH profile on metabolism and cancer risk [203].

Side effects of rhGH treatment at the recommended doses are usually mild and well tolerated and are generally reversible by decreasing the dose of GH. The most common adverse reactions are injection site discomfort, edema, paresthesia, joint pain, and carpal tunnel syndrome. In addition, hyperglycemia or diabetes mellitus may develop as a result of the hyperglycemic effect of GH, especially in obese patients [204]. Treatment with rhGH is contraindicated in patients with active cancer or in those with conditions that could be exacerbated by increased cell growth [197].

### 5.5. Arginine Vasopressin Deficiency

The aim of the treatment of AVP deficiency is to control the polyuria–polydipsia syndrome and prevent damage caused by osmolar disturbances. To achieve this goal, it is necessary to ensure adequate fluid intake, provide hormone replacement therapy, and correct any associated hydroelectrolytic and hemodynamic disturbances [205,206].

It is essential to ensure adequate fluid intake to prevent dehydration, especially in patients with significant polyuria. Patients should be educated about the importance of adequate fluid intake. The recommended amount of water to drink per day may vary depending on several factors, including age, gender, level of physical activity, climate, and overall health of the individual.

Because AVP has a short plasma half-life of 5 to 10 min, it is not suitable for hormone replacement therapy. Therefore, the analog deamino D-AVP (desmopressin, DDAVP) is used, which has a half-life of 6 to 8 h and lacks the pressor effect of AVP [207,208].

DDAVP can be administered orally (standard tablets and sublingual preparations), intranasally (via rhinal tube or dose spray), and parenterally (subcutaneously or intravenously), depending on the patient’s needs and clinical presentation.

In general, the oral route of administration is preferred to the intranasal route because it offers greater bioavailability and less variability. Daily doses of standard tablets and sublingual tablets range from 100–400 µg and 60–240 µg, respectively, divided into doses and administered every 8 to 12 h. DDAVP dose titration should be performed on the basis of its clinical effect (control of thirst and polyuria), plasma and urine osmolality, and natremia [206].

Intranasal DDAVP has a wide intra- and interindividual variability in bioavailability and half-life, which affects its efficacy. In addition, its efficacy is also reduced in the presence of congestion, inflammation, or scarring of the nasal mucosa, and it is not suitable for use after transsphenoidal surgery. In addition, the use of nasal formulations is associated with an increased risk of administration errors and higher rates of iatrogenic hyponatremia. The dose of intranasal DDAVP varies from 10 to 20 µg/day once or twice daily as needed [116,206].

DDAVP is usually administered parenterally (subcutaneously or intravenously) perioperatively in transsphenoidal surgery or after TBI [205]. To avoid iatrogenic hyponatremia, it should be noted that a higher dose of parenteral DDAVP not only increases the magnitude of the antidiuretic response, but also prolongs the time during which this response is maintained. For example, a dose of 1 µg intravenously can prolong the clinically significant effect to approximately 12 h, whereas a dose of 8 µg can prolong the clinically significant effect to 48 h [209].

### 5.6. Oxytocin Deficiency

Currently, oxytocin is indicated for intravenous administration to induce or stimulate uterine contractions in case of dynamic dystocia [210] and is used intramuscularly or intravenously to prevent and treat postpartum hemorrhage [211].

On the other hand, it is well known that patients with HP often experience decreased quality of life, accompanied by increased morbidity and mortality, even with optimal hormone replacement therapy [88]. This is particularly evident in patients with craniopharyngioma, where a significant impact on emotional and social function has been observed [212,213,214]. Recent research suggests that patients with HP, particularly those with AVP deficiency, may also have an associated oxytocin deficiency, which may contribute to alterations in cognitive empathy [146].

Preliminary data suggest that oxytocin therapy may be beneficial in improving cognitive and metabolic problems associated with HP and craniopharyngioma. Intranasal administration of oxytocin has shown the potential to improve certain neuropsychological and metabolic deficits in these patients [215]. However, before oxytocin can be considered a viable treatment option for HP, further studies are needed to clearly establish the diagnosis of oxytocin deficiency and to adequately determine the optimal dose, frequency, and route of administration. In addition, the best form of follow-up and the knowledge of long-term safety need to be established in order to ensure the best clinical outcomes.

## 6. Interactions between Hormone Replacement Therapies in Hipopituitarism

Interactions between hormone replacement therapies in HP are a fundamental aspect to take into account in the management of this complex endocrine condition. The simultaneous administration of different hormones can result in cross effects that affect the efficacy of treatment and the overall health of the patient. Understanding these interactions is critical to optimizing therapy and minimizing risks, thus ensuring a comprehensive and personalized approach to the care of patients with HP. Table 4 shows different clinical considerations on the interactions between different hormone replacement therapies in HP.

### 6.1. Glucocorticoids

The hypothalamic–pituitary–adrenal axis influences water balance through the stimulatory effects of CRH and the inhibitory effects of cortisol on AVP secretion, as well as through the peripheral effects of cortisol on hemodynamics and renal water handling [216,217,218]. Therefore, the clinical presentation of a deficiency of AVP may be masked by adrenal insufficiency and uncovered after initiation of hormone replacement therapy with glucocorticoids [219].

### 6.2. Levothyroxine

Thyroid hormones increase the activity of the liver enzymes that metabolize cortisol. As a result, administration of LT4 may increase the rate of cortisol metabolism, resulting in a decrease in blood cortisol levels. Therefore, in patients with hypothalamic–pituitary disorders where ACTH and cortisol deficiencies may be present, it is critical to evaluate and treat adrenal insufficiency prior to initiating LT4 therapy. If this evaluation is not possible, empiric hydrocortisone therapy is recommended until the adrenal axis can be properly assessed [220,221].

### 6.3. Sex Hormones

Oral estrogens, via the first-pass metabolism in the liver, have a potent effect on the synthesis of various proteins, including hormone transport proteins like cortisol-binding globulin (CBG) and thyroxine-binding globulin (TBG). However, this effect is not observed with transdermal estrogens [222]. Therefore, when assessing the hypothalamic–pituitary–adrenal axis, it should be taken into account that the circulating levels of total serum cortisol may be elevated due to the effects of oral estrogen on CBG.

On the other hand, the increase in circulating TBG levels induced by oral estrogens may increase the bound fraction (total T4) and decrease the free (bioactive) fraction of circulating T4 (FT4), which may increase the need for LT4 in hypothyroid women on hormone replacement therapy [222,223]. Therefore, it is advisable to monitor FT4 levels to adjust the doses of hormone replacement therapy with LT4 after initiating oral estrogen therapy.

Oral estrogen therapy has been shown to reduce circulating IGF-1 levels in women with GHD receiving hormone replacement therapy with rhGH. However, this effect is not observed with transdermal estrogen preparations. This suggests that the hepatic first-pass effect of oral estrogens is associated with a resistance to GH action in the liver, implying the need to increase the rhGH dose to maintain circulating IGF-1 levels within the age-adjusted normal range [224,225]. Therefore, a non-oral route of estrogen replacement is recommended whenever possible to optimize the cost–benefit ratio of GH replacement in GHD women [225].

### 6.4. Growth Hormone

rhGH therapy may significantly affect circulating hormone levels and the required doses of other hormone replacement therapies [226,227,228,229].

GH, through its mediator IGF-I, inhibits the activity of the 11β-hydroxysteroid dehydrogenase type 1 enzyme that converts the inactive metabolite cortisone into cortisol (the active form of the glucocorticoid). Therefore, rhGH therapy can decrease cortisol bioavailability, which can “unmask” partial ACTH deficiency and lead to acute adrenal insufficiency or adrenal crisis if glucocorticoid doses are not adequately adjusted [230].

rhGH therapy also affects thyroid hormone metabolism by stimulating the activity of type 2 deiodinase, an enzyme that converts T4 to triiodothyronine (T3). As a result of this increased conversion, FT4 may decrease, so it may be necessary to initiate or increase the dose of LT4 replacement therapy in patients with central or primary hypothyroidism [231,232,233].

GH has anti-insulin effects that may increase insulin resistance and consequently increase blood glucose levels. Therefore, it is important to monitor blood glucose levels in patients without a history of diabetes, and in patients with diabetes, it may be necessary to adjust the therapeutic regimen to maintain adequate control of blood glucose levels [234,235,236].

## 7. Conclusions and Recommendations for Future Research

The present review of HP highlights the complexity of this endocrine disorder, which can result from a variety of etiologies, including pituitary tumors, hypothalamic lesions, and genetic factors. An early and accurate diagnosis is fundamental to the effective management of HP, allowing timely initiation of hormone replacement therapy, which is critical to improving patients’ quality of life. Furthermore, the importance of a multidisciplinary approach to treatment is emphasized, given the significant impact this condition can have on long-term morbidity and mortality.

In the future, research should be carried out focusing on different aspects, including the following: (1) research in pathophysiology, which would deepen the understanding of the pathophysiological mechanisms that lead to HP and could open new avenues for its prevention and treatment; (2) the development of new biomarkers, investigating and validating those that allow early diagnosis and assessment of pituitary function, which would facilitate faster and more effective intervention; (3) the exploration of new therapeutic strategies, including innovative treatments that go beyond hormone replacement therapy, and personalized approaches based on the patient’s genetics and molecular biology; (4) conducting longitudinal studies assessing the long-term impact of HP on quality of life, mental health, and associated comorbidities to improve management strategies; and (5) assessing the psychosocial impact, investigating the effects of HP on patients and their families in order to develop support programs and resources that address their emotional and social needs.

## Figures and Tables

**Table 1:** Demographic and etiologic distribution of cases of hypopituitarism according to the main published series.

Author, Year	Ref.	n	Gender Male, n (%)	Age (yr)	Tumor Etiology n (%)		Non-Tumor Etiology n (%)
					Pituitary Tumors	Non-Pituitary Tumors	
Regal et al., 2001	[1]	69	32 (46.4)	50.0 ± 17.0	42 (61)	6 (9)	21 (30)
Tanriverdi et al., 2014	[4]	773	385 (49.8)	43.9 ± 16.1	337 (43.6)	56 (7.2)	380 (49.2)
Doknic et al., 2017	[5]	512	297 (57.9)	45.9 ± 1.7	297 (57.9)	43 (8.4)	172 (33.6)
Total		1354	714 (52.7)	46.6 ± 11.6	676 (49.9)	105 (7.7)	573 (42.3)

**Table 2:** Clinical manifestations and diagnostic approaches for hormonal deficiencies in adult patients with hypopituitarism.

Hormonal Deficiency	Clinical Symptoms	Diagnostic Tests
ACTH	Fatigue, muscle weakness, weight loss, hypoglycemia, hyponatremia	Normal/low plasma ACTH Serum cortisol (08:00–09:00 a.m. and at least 18–24 h after last dose of hydrocortisone): <3 mcg/dL → Confirm AI >15 mcg/dL → Rule out AI 3–15 mcg/dL → Stimulations testing: ➢ ITT: 0.1 U/kg iv → Gly < 40 mg/dL → Normal response: peak cortisol > 18 mcg/dL ➢ ACTH test: 250 mcg iv → Normal response: peak cortisol ≥ 18 mcg/dL
TSH	Fatigue, weakness, weight gain, dry skin, cold intolerance, depression	Normal/low serum TSHDecreased serum free T4
FSH/LH	Micropenis, cryptorchidism, and micro-orchidism in the male neonate; no phenotype in the female neonate Delayed/absent puberty in both sexes Amenorrhea, oligomenorrhea, loss of libido, vaginal dryness, hot flashes, breast atrophy (in women); erectile dysfunction, decreased libido, muscle loss, testicular atrophy (in men)	Normal/low serum FSH and LH Decreased serum 17-beta-estradiol (women) Decreased total serum testosterone (men)
GH	Slow growth in children, changes in body composition in adults, fatigue, depression	Normal/low serum IGF-1 GH stimulation tests ➢ ITT: 0.1 U/kg iv → Gly < 40 mg/dL → Severe GHD: peak GH < 3 ng/mL ➢ Glucagon 1 mg im → Severe GHD: peak GH < 3 ng/mL
Prolactin	Inability to lactate (agalactia)	Low serum PRL
AVP	Polyuria, polydipsia, dehydration, confusion, irritability	Measurement of urine osmolality and volume; response to desmopressin administration (>9–50% increase) Unstimulated plasma levels of copeptin ≤21.4 pmol/L → copeptin stimulation tests Post-stimulation copeptin level ➢ ≤4.9 pmol/L after hypertonic saline ➢ ≤3 pmol/L after administration of arginine
Oxytocin	Difficulties in lactation	Clinical evaluation; no widely accepted standardized diagnostic tests for oxytocin

Abbreviations: AI, adrenal insufficiency; AVP, arginine vasopressin; GHD, growth hormone deficiency; Gly, glycemia; ITT, insulin-induced hypoglycemia test (insulin tolerance test).

**Table 3:** Therapeutic interventions for hormonal deficiencies in adult patients with hypopituitarism.

Hormonal Deficiency	Treatment
ACTH	Glucocorticoids Hydrocortisone is the preferred treatment, typically administered in divided doses to mimic the natural circadian rhythm of cortisol. The usual total daily dose ranges from 10 to 20 mg, with adjustments made during periods of stress (e.g., infections, surgeries), when requirements may increase significantly.
TSH	Levothyroxine (LT4) Administered orally is the standard treatment. The dosage is individualized based on serum free T4 levels, aiming to maintain in the upper half of the normal range. Regular monitoring is essential to adjust the dose as needed.
FSH/LH	Women Estrogen and progesterone therapy to manage symptoms of menopause and maintain secondary sexual characteristics. Men Treatment with gonadotropins during the neonatal period to induce an increase in penile length and testicular size. In some cases, gonadotropin therapy may help induce testicular descent prior to surgery. Treatment with gonadotropins or testosterone to induce puberty and promote the development of secondary sexual characteristics. Testosterone replacement therapy administered via injections, patches, or gels to restore testosterone levels and alleviate symptoms such as erectile dysfunction and decreased libido.
GH	rhGH Administered via subcutaneous injection, this therapy is tailored to the individual’s needs, with doses adjusted based on growth response in children or body composition changes in adults. Regular monitoring of IGF-1 levels is necessary to ensure efficacy and safety.
PRL	No hormone replacement therapy is available.
AVP	Desmopressin (DDAVP) This synthetic analog of AVP can be administered intranasally, orally, or by iv, depending on the patient’s needs. Dosage is individualized based on response and urine output.
Oxytocin	Oxytocin Further studies are needed to understand long-term safety and to adequately determine the optimal dose, frequency, and route of administration.

**Table 4:** Considerations on interactions between different hormone replacement therapies in hypopituitarism.

Hormone Replacement Therapy	Potential Interaction	Clinical Effect	Recommendations
Glucocorticoids (GCs)	GCs play a permissive role in the renal excretion of free water and have inhibitory effects on AVP secretion.	AI may mask AVP deficiency, and GC therapy may highlight the clinical presentation of AVP deficiency.	Consider that GC treatment may unmask the presence of a covert AVP deficiency.
Levothyroxine (LT4)	Increases the activity of liver enzymes that metabolize cortisol.	May decrease cortisol levels in patients with ACTH deficiency.	Monitor cortisol levels and consider hydrocortisone therapy before starting LT4.
Estrogens	Oral estrogens increase CBG synthesis.	It does not necessarily reflect an increase in the free and bioactive fraction of cortisol.	Avoid overestimation of total cortisol levels.
	Oral estrogens increase TBG synthesis.	May increase total T4 and decrease FT4 levels, increasing the need for LT4 in hypothyroid women treated with LT4.	Monitor FT4 levels to adjust the doses.
	Oral estrogens may reduce circulating IGF-1 levels in patients receiving rhGH.	Can lead to resistance to GH action in the liver, necessitating higher doses of rhGH to maintain IGF-1 levels within the normal range.	Monitor IGF-1 levels regularly and consider increasing the dose of rhGH if levels fall below the target range.
rhGH	Decreases cortisol bioavailability by inhibiting the activity of the enzyme 11β-hydroxysteroid dehydrogenase type 1 that converts cortisone to cortisol.	Can “unmask” partial ACTH deficiency, leading to acute AI if glucocorticoid doses are not adjusted.	Monitor cortisol levels closely and adjust glucocorticoid therapy as needed to prevent adrenal crisis.
	Stimulates the activity of type 2 deiodinase.	Increases the conversion of T4 to T3, decreasing FT4 levels.	Monitor T4 levels regularly and adjust LT4 dosage as necessary.
	Increases insulin resistance.	Increases the risk of hyperglycemia or diabetes.	Monitor blood glucose levels regularly and adjust diabetes management as necessary.

Abbreviations: AI, adrenal insufficiency; CBG, cortisol-binding globulin; FT3, free triiodothyronine; FT4, free thyroxine; GC, glucocorticoids; LT4, levothyroxine; rhGH, recombinant human growth hormone; TBG, thyroxine-binding globulin; T4, thyroxine.
